# A Genome-Wide Association study in *Arabidopsis thaliana* to decipher the adaptive genetics of quantitative disease resistance in a native heterogeneous environment

**DOI:** 10.1371/journal.pone.0274561

**Published:** 2022-10-03

**Authors:** Fabrice Roux, Léa Frachon

**Affiliations:** 1 Laboratoire des Interactions Plantes-Microbes-Environnement, Institut National de Recherche pour l’Agriculture, l’Alimentation et l’Environnement, CNRS, Université de Toulouse, Castanet-Tolosan, France; 2 Department of Systematic and Evolutionary Botany, University of Zürich, Zürich, Switzerland; Kansas State University, UNITED STATES

## Abstract

Pathogens are often the main selective agents acting in plant communities, thereby influencing the distribution of polymorphism at loci affecting resistance within and among natural plant populations. In addition, the outcome of plant-pathogen interactions can be drastically affected by abiotic and biotic factors at different spatial and temporal grains. The characterization of the adaptive genetic architecture of disease resistance in native heterogeneous environments is however still missing. In this study, we conducted an *in situ* Genome-Wide Association study in the spatially heterogeneous native habitat of a highly genetically polymorphic local mapping population of *Arabidopsis thaliana*, to unravel the adaptive genetic architecture of quantitative disease resistance. Disease resistance largely differed among three native soils and was affected by the presence of the grass *Poa annua*. The observation of strong crossing reactions norms among the 195 *A*. *thaliana* genotypes for disease resistance among micro-habitats, combined with a negative fecundity-disease resistance relationship in each micro-habitat, suggest that alternative local genotypes of *A*. *thaliana* are favored under contrasting environmental conditions at the scale of few meters. A complex genetic architecture was detected for disease resistance and fecundity. However, only few QTLs were common between these two traits. Heterogeneous selection in this local population should therefore promote the maintenance of polymorphism at only few candidate resistance genes.

## Introduction

During their life cycle, plants are simultaneously and/or sequentially challenged by multiple pathogens, whether in crop fields or in wild habitats [1]. Pathogens are widely recognized as one of the major selective agents in nature, thereby influencing the eco-evolutionary trajectories of natural plant populations [2]. In particular, pathogens can influence the distribution of polymorphism at loci affecting plant resistance, which may in turn affect the patterns of disease incidence, prevalence and evolution [3]. Disease resistance is highly diverse both within and among plant populations, providing the opportunity to study plant-pathogen coevolution [2, 4]. Most theoretical papers on plant-pathogen coevolution focus on qualitative resistance (presence/absence of symptoms), which is related to the gene-for-gene (GFG) relationship [3]. However, quantitative disease resistance (QDR, continuum of symptoms) is much more prevalent than qualitative resistance in natural plant populations [1, 5, 6]. In line with this observation, high-throughput analyses combined with systems biology approaches revealed that plant immunity corresponds to a decentralized (i.e. not centered on a specific hub) and highly connected molecular network rather than to the simplistic view of two layers of the immune system (namely pathogen-associated molecular pattern (PAMP)-triggered immunity (PTI) and effector triggered immunity (ETI)) [7].

Predictions of co-evolutionary dynamics in plant pathosystems differ between quantitative resistance and qualitative resistance [8, 9]. In particular, it is hypothesized that the emergence of a new disease would first lead to coevolution of quantitative host resistance and pathogen virulence, which would result in the stabilization of allele frequencies at multiple genes associated with host immunity and pathogen virulence over short co-evolutionary times [10]. Over longer co-evolutionary periods, this stabilization would shift to long-period cycles in the frequency of qualitative resistance and qualitative virulence, leaving signatures of balancing selection on underlying genes, as predicted by the GFG model [10, 11].

Besides the complexity of the genetic architecture of plant-pathogen interactions that has been recently demonstrated by joint genome-wide association studies (GWAS) [12, 13], host-pathogen co-evolutionary dynamics can be dramatically impacted by the environment [14–17]. Numerous experimental studies highlighted the effect of abiotic and biotic factors on the outcome of wild plant-pathogen interactions at different spatial and temporal grains, with the extreme case of genotypes of plants and pathogens switching ranks between environments for resistance and virulence, respectively [18–22]. Such crossing reaction norms in heterogeneous environments are thought to delay fixation of a given resistance strategy, thereby constraining co-evolutionary dynamics and potentially favoring the maintenance of genetic variation of plant resistance in natural plant populations [3, 16].

Studies on the effects of the type of resistance (qualitative *vs* quantitative) and heterogeneous environments on co-evolutionary dynamics provided a solid ground in our understanding of plant-pathogen interactions. However, studies reporting the adaptive genetic architecture of quantitative disease resistance in a heterogeneous environment remains scarce. In this study, we set up an *in situ* GWAS to fine map Quantitative Trait Loci (QTLs) associated with both disease resistance and fecundity using the local mapping population TOU-A of the highly selfing species *Arabidopsis thaliana* [23], which is located in a heterogeneous abiotic and biotic environment [24]. *A*. *thaliana* inhabits contrasting environments for diverse abiotic (e.g. climate, soil physico-chemical properties) and biotic (e.g. microbial communities, plant communities) factors [24–29], with disease incidence being common in its natural populations [1, 30, 31]. For instance, in a survey of 163 natural populations in south-west of France, 72.7% of plants presented disease symptoms and each of the two most abundant bacterial pathogenic species (namely *Pseudomonas viridiflava* and *Xanthomonas campestris*) was detected by a metabarcoding approach in more than 90% of natural populations [27].

Specifically, we aimed at addressing the following questions: 1) What is the extent of natural genetic variation in disease resistance among 195 local accessions scored in their native habitat?, 2) How is genetic variation in disease resistance spatially distributed within the local population?, 3) Does genetic variation for disease resistance present signatures of natural selection?, 4) What is the genetic architecture of disease resistance in a local population?, 5) Do QTLs associated with disease resistance overlap with QTLs associated with fecundity?, 6) Do the answers to the five previous questions depend on the natural soil agronomic properties and/or the presence of a co-occurring plant species in the local TOU-A population?

## Results

### Natural genetic variation in disease index and crossing reaction norms

We first aimed at estimating the extent of natural genetic variation for disease resistance among local genotypes scored in contrasting micro-habitats and test whether the ranking of accessions for disease resistance shifted among micro-habitats. In this study, we focused on 195 whole-genome sequenced accessions that have been collected along a transect of 350-m in the highly genetically polymorphic local population TOU-A (France, Burgundy, 46° 38′ 57.302″ N, 4°7′ 16.892″ E) located under an electric fence separating two permanent meadows experiences cycles of grazing by cattle [24]. The mean distance between consecutive collected accessions was 1.67m (min = 0m, max = 41.8m, confidence intervals 95% = 10 cm– 8.1m). The 195 accessions were grown *in situ* within the TOU-A population in six micro-habitats [24], which consist of three soil types crossed with the presence or absence of the bluegrass *Poa annua*, a species frequently associated with *A*. *thaliana* in natural habitats [24, 32, 33]. Based on chemical analyses of 14 edaphic factors for 83 samples collected along the transect [24], we determined three soil types (hereafter named soils A, B and C) that were naturally contrasted for their agronomic properties. Soil A has a higher pH, higher concentrations of nitrogen, calcium, magnesium and a lower concentration of aluminum than soil C, soil B having intermediate values for these five edaphic factors between soils A and C (S1 Fig).

Within each of the six micro-habitats, we grew five replicates of each accession in a randomized complete block design with one replicate per block. Each block was represented by three arrays of 66 individual wells, with 195 wells that were sown with seeds and three remaining wells that were not sown with seeds. All the wells were first filled with 3 cm of the respective native soil, then with an additional 1cm of the respective native soil that was oven dried for two days at 65°C. Germination from the seed bank was therefore prevented by the oven dried native soil. To mimic the main natural germination cohort observed in the TOU-A population, seeds were sown directly *in situ* on the three native soils A, B and C in late September 2012. For each natural soil, we manipulated the presence of *P*. *annua* by sowing five seeds of *P*. *annua* in each well and then thinning them to obtain only one *P*. *annua* plant per well [24].

**Fig 1:**
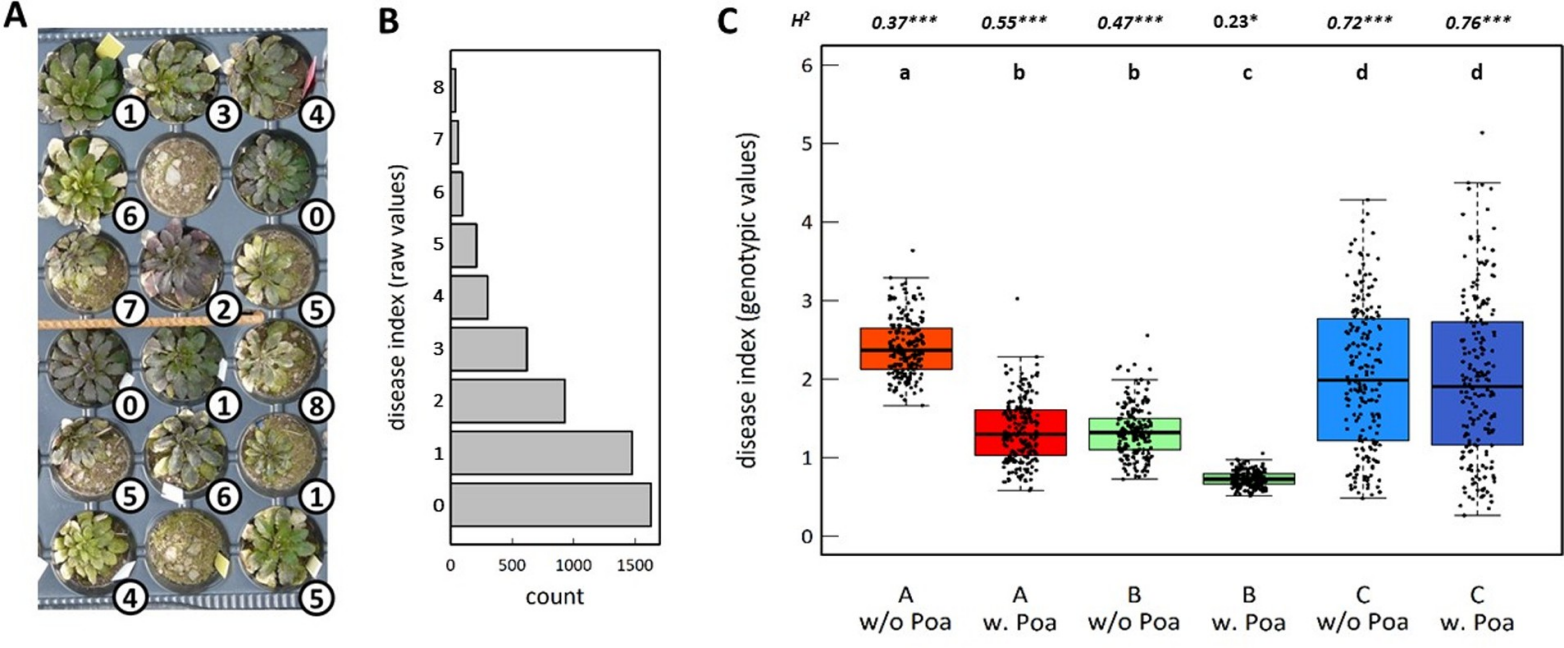
Genetic variation among the 195 TOU-A accessions for disease index across the six micro-habitats. (A) Photograph illustrating the observed variation in the level of disease index ranking from zero to eight. Each value on the bottom right of each plant indicates the level of disease index scored on March 3^rd^, 2013. (B) Variation of disease index across the 5,367 plants scored in this study. (C) Genetic variation within each micro-habitat. Each dot corresponds to the genotypic values (Best Linear Unbiased Estimator) for disease index of one of the 195 accessions. ‘A w/o Poa’: soil A in absence of *Poa annua*, ‘A w. Poa’: soil A in presence *Poa annua*, ‘B w/o Poa’: soil B in absence of *Poa annua*, ‘B w. Poa’: soil B in presence *Poa annua*, ‘C w/o Poa’: soil C in absence of *Poa annua*, ‘C w. Poa’: soil C in presence *Poa annua*. Different upper letters indicate different groups according to the micro-habitat after a Tukey correction for multiple pairwise comparisons. *H*²: broad-sense heritability value for each microhabitat. Italic values indicate statistically significant results after a Bonferroni correction for multiple comparisons. *** *P* < 0.001, * *P* < 0.05.

After overwintering at the rosette stage, 5,367 plants were scored early March 2013 for disease symptoms in a semi-quantitative manner with a scale ranging from zero to ten. These eleven scores categorize the percentage of rosette area infected ranging from 0% (absence of visible symptoms) to 100% (visible symptoms on the entire rosette area), with an increment of 10%. The symptoms considered in this study were determined by the presence of visible chlorosis, visible necrosis, leaf mosaic or water-soaked lesions and cell death related symptoms surrounding infection sites. Genetic variation for this set of symptoms have been observed in *A*. *thaliana* in response to diverse pathogens, either viruses, bacteria, fungi and/or oomycetes, in either greenhouse, field or natural conditions [34–40] (Fig 1A). In addition, the presence of such disease symptoms was associated with a significantly higher relative proportion of the pathobiota in the leaf bacterial microbiota in natural populations of *A*. *thaliana* [27].

Two-thirds of the plants presented disease symptoms (Fig 1B). While most diseased plants presented few symptoms (disease index = 1, n = 1,476, 39.5%, Dataset 1 in S1 Dataset), a non-negligible fraction of diseased plants presented severe symptoms (disease index > = 5, n = 407, 10.9%, Dataset 1 in S1 Dataset) (Fig 1B). Because resources involved in plant development may not be further available for plant immunity, we investigated this trade-off [41, 42] by testing whether plant development affected disease resistance. In line with the growth-immunity trade-off, disease index was significantly and positively correlated with plant size (i.e. maximal rosette diameter used as a proxy) scored before the onset of winter (Table 1 and S2 Fig). However, variation in rosette size explained on average only 2.9% of disease index variation across the six micro-habitats. Disease index was not different between plants that bolted before (n = 2,608, 48.6%) or after (n = 2,759, 51.4%) early March 2013 (Table 1).

**Table 1:** Genetic variation among the 195 TOU-A accessions for disease index across the six micro-habitats. Model terms with parentheses indicate nested effects (see Model 1). Random effects are in italic. Model random terms were tested with likelihood ratio tests of models with and without these effects following a chi-square distribution with a degree of freedom of 1.

Model terms	*F* or λ_LR_	*P*
block(soil*comp)	21.0	**1.0 E-32**
soil	29.6	**1.7E-13**
comp	1.1	3.0E-01
soil*comp	10.2	**3.7E-05**
*Acc*	39.4	**3.5E-10**
Acc*soil	79.2	**1.0 E-16**
Acc*comp	0.0	1.0E+00
Acc*soil*comp	8.7	**3.2E-03**
bolting (soil*comp)	2.6	1.7E-02
rosette diameter (soil*comp)	18.4	**3.0E-21**

Bold *P*-values indicate significant effect after Bonferroni correction. λ_LR_: likelihood-ratio test statistic. ‘soil’: soils A, B and C. ‘comp’: absence or presence of *P*. *annua*. ‘Acc’: accession. ‘Bolting’: binary trait corresponding to the presence or absence of an inflorescence distinguishable from the leaves, on the day plants were scored for disease symptoms. ‘Rosette diameter’: maximal rosette diameter before the onset of winter.

**Fig 2:**
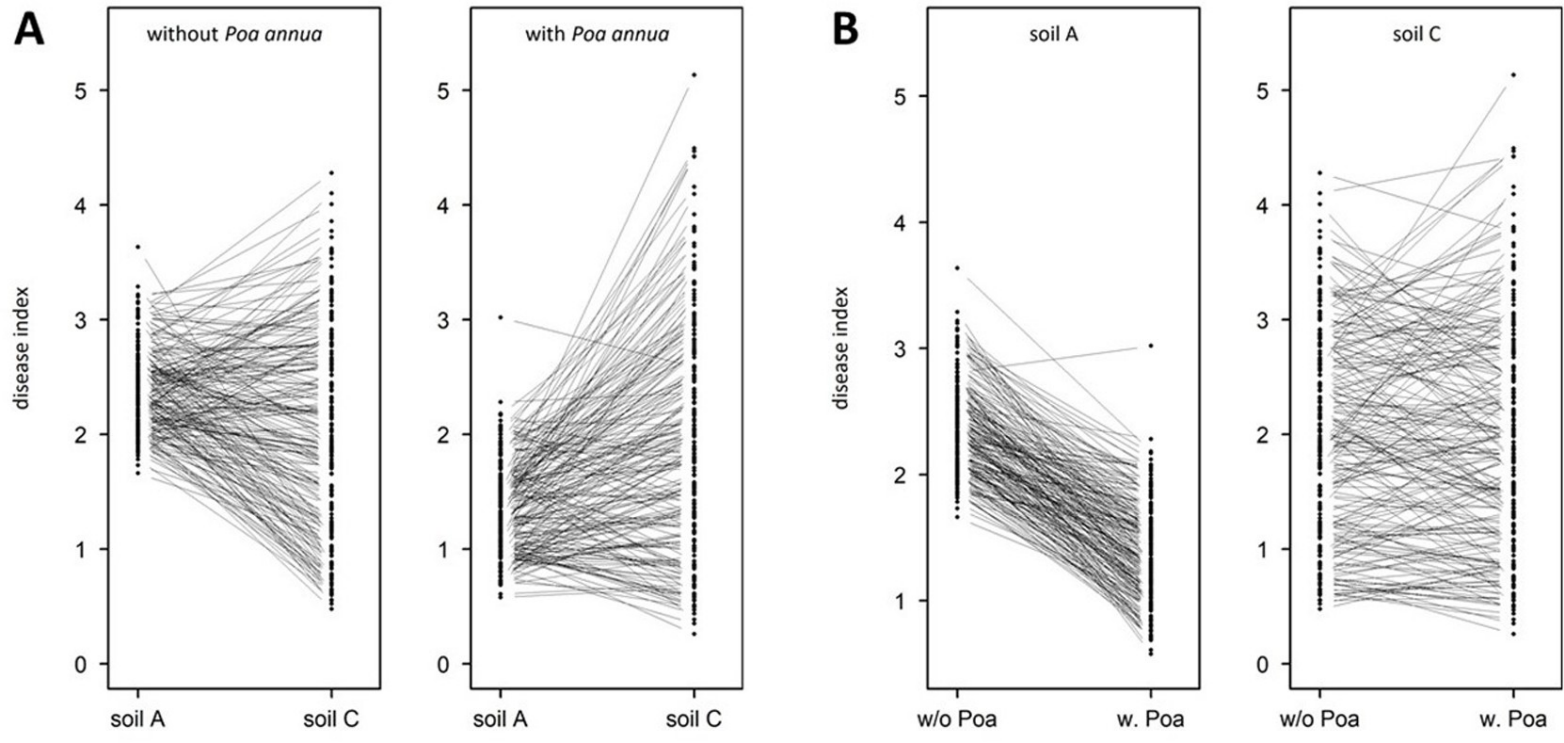
Extensive Genotype x Environment (GxE) interactions across the micro-habitats. (A) GxE interactions between soil A and soil C in absence (left panel) and presence (right panel) of *P*. *annua*. (B) GxE interactions between the absence and presence of *P*. *annua* on soils A (left panel) and C (right panel). Each dot corresponds to the Best Linear Unbiased Estimator (BLUE) for disease index of one of the 195 accessions. Each line connects one of the 195 accessions between two micro-habitats.

The mean disease index largely differed among the six micro-habitats, with a significant interacting effect between the soil type and the presence/absence of *P*. *annua* (Table 1). In absence of *P*. *annua*, plants presented on average more disease symptoms in soil A than in soil B, plants in soil C presenting significantly intermediate levels of disease symptoms (Fig 1C). The presence of *P*. *annua* significantly reduced the level of disease symptoms in soils A and B, but not in soil C (Fig 1C). Extensive genetic variation was found among the 195 accessions in each micro-habitat (in particular in soil C), with the exception of soil B in presence of *P*. *annua* for which no significant genetic variations was detected (Tables 1 and S1 and Fig 1C). Because the absence of genetic variation among accessions precludes any genotypic selection analyses and GWA mapping, the micro-habitat ‘soil B in presence of *P*. *annua*’ was not further considered in the rest of the study. Among the five remaining micro-habitats, broad-sense heritability values ranged from 0.37 to 0.76 (Fig 1C). Highly significant ‘accessions × soil type’ and ‘accession × soil type × presence/absence of *P*. *annua*’ interactions combined with the observation of strong crossing reaction norms, indicate that the ranking of accessions for disease index was different among the micro-habitats (Table 1). These extensive Genotype x Environment (GxE) interactions are well illustrated by comparing genotypic values of disease index between soils A and C (both in absence and presence of *P*. *annua*, Fig 2A) as well as between the absence and presence of *P*. *annua*, in particular in soil C (Fig 2B).

### Spatial genetic variation in disease index

After detecting highly significant genetic variation in disease index in five out of the six micro-habitats tested, we aimed at estimating the degree of patchiness of this genetic variation along the 350-m environmentally heterogeneous transect. To estimate the spatial grain of disease index variation, we first performed a spectral decomposition of the spatial relationships among the 195 accessions. We identified 59 Principal Coordinates of Neighbor Matrices (PCNM) components, suggesting a relatively homogeneous spatial distribution of the 195 accessions along the 350-m transect (S3 Fig), which is in line with the mean distance between consecutive collected accessions of 1.67m. The spatial grain of disease index largely differed among the micro-habitats. While a coarse-grained spatial variation was detected in soil A in absence of *P*. *annua*, a fine-grained spatial variation was detected in soil C both in absence and presence of *P*. *annua* (S2 Table). No significant spatial variation was detected in soil A in presence of *P*. *annua* and in soil B in absence of *P*. *annua*, suggesting a random spatial distribution of disease index variation scored in these two micro-habitats (S2 Table).

### Fecundity-disease index relationship estimated by a genotypic selection analysis

**Fig 3:**
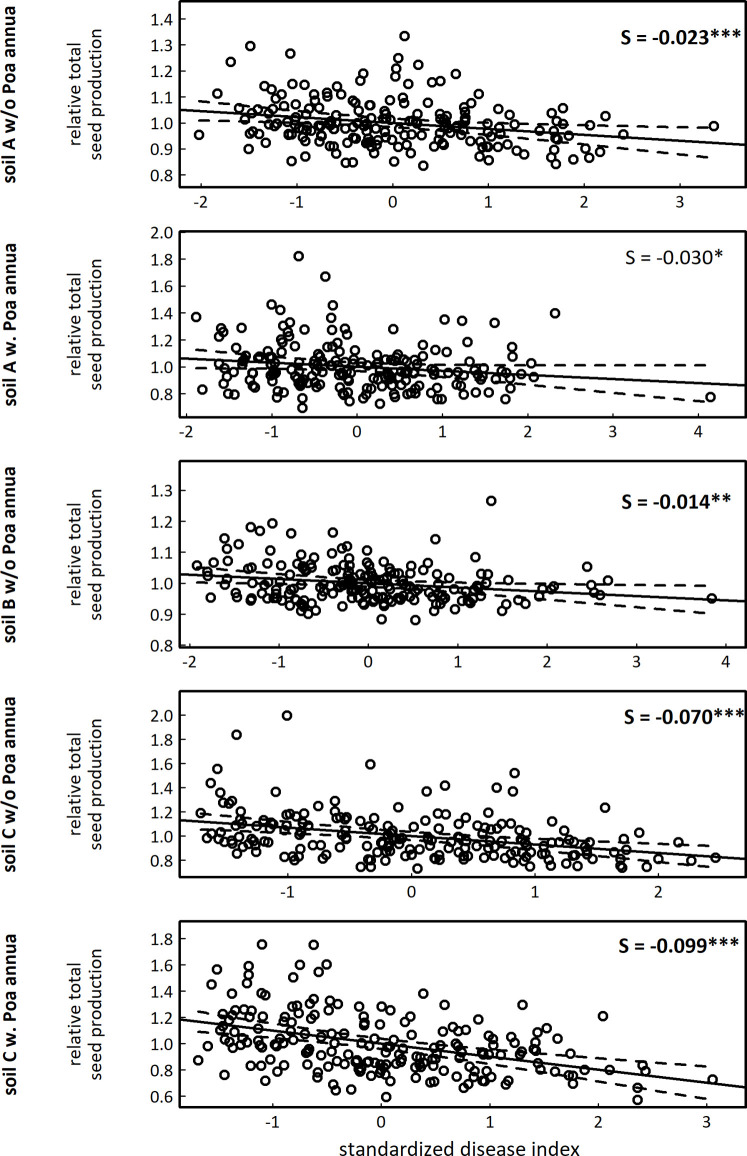
Genotypic selection analysis between relative total seed production and standardized disease index with selection differential (S) within each micro-habitat for which disease index was significantly heritable. Each dot corresponds to the Best Linear Unbiased Estimator (BLUE) for disease index of one of the 195 accessions. ‘w/o’: absence of *P*. *annua*, ‘w.’: presence of *P*. *annua*. The solid line corresponds to the fitted regression line, whereas the dashed lines delimit the band of 99% confidence intervals. *** *P* < 0.001, ** *P* < 0.01, * *P* < 0.05. Bold *P* values indicate statistically significant results after a Bonferroni correction for multiple comparisons. Note that the scale for the *x* and *y* axes are different among the five micro-habitats.

As a next step, we aimed at testing whether genetic variation in disease index presented signatures of natural selection. To do so, we performed a genotypic selection analysis by estimating the selection differential S, which is a measure of association between trait values and fitness estimates [43, 44]. Negative S values predict that natural selection would favor genotypes with low values for the phenotypic trait of interest. Fitness estimate is usually approximated by the total number of seeds produced by a plant, which has been demonstrated as a good proxy of fecundity in highly selfing species such as *A*. *thaliana* [25, 45]. Because the number of seeds per fruit is highly correlated with fruit length in *A*. *thaliana* [46], total seed production of each plant scored for disease index was approximated by measuring total silique length [24]. Based on genotypic values (Dataset 2 in S1 Dataset), we first calculated relative fecundity and standardized disease index in each micro-habitat for which disease index was significantly heritable. We found standardized S estimates to be negative across micro-habitats, indicating that accessions presenting severe symptoms produced on average fewer seeds than accessions with few symptoms (Fig 3). In addition, S estimates were significantly different among the three soil types (S3 Table), with S estimates in soil C being between 2.3 and 7.1 higher than S estimates in soils A and B (Fig 3). The presence of *P*. *annua* did not significantly affect S estimates (S3 Table and Fig 3).

### Genetic architecture of disease index and fecundity

As a last step, we aimed at describing the genetic architecture underlying natural genetic variation in disease index and estimating the percentage of detected QTLs that were also associated with natural genetic variation of fecundity. To do so, we set up GWA mapping analyses by taking advantage of the genome sequencing of the 195 TOU-A accessions, which revealed a set of 1,902,592 Single Nuclear Polymorphisms (SNPs) and a linkage disequilibrium (LD) decay to *r*² = 0.5 within an average of 18 base pairs [24]. To fine map QTLs associated with natural variation of disease index down to the gene level, we combined a mixed-model approach correcting for the effects of population structure with a local score approach, the latter approach allowing delimiting QTL regions by accumulating single marker *p*-values obtained from the mixed-model while controlling the issue of multiple hypothesis tests [47]. The efficiency of this combination was demonstrated in the TOU-A population, with the fine mapping (in particular, the detection of QTLs with small effects) and the cloning of four of the QTLs associated with quantitative disease resistance to the bacterial pathogen *Ralstonia solanacearum* [48].

**Fig 4:**
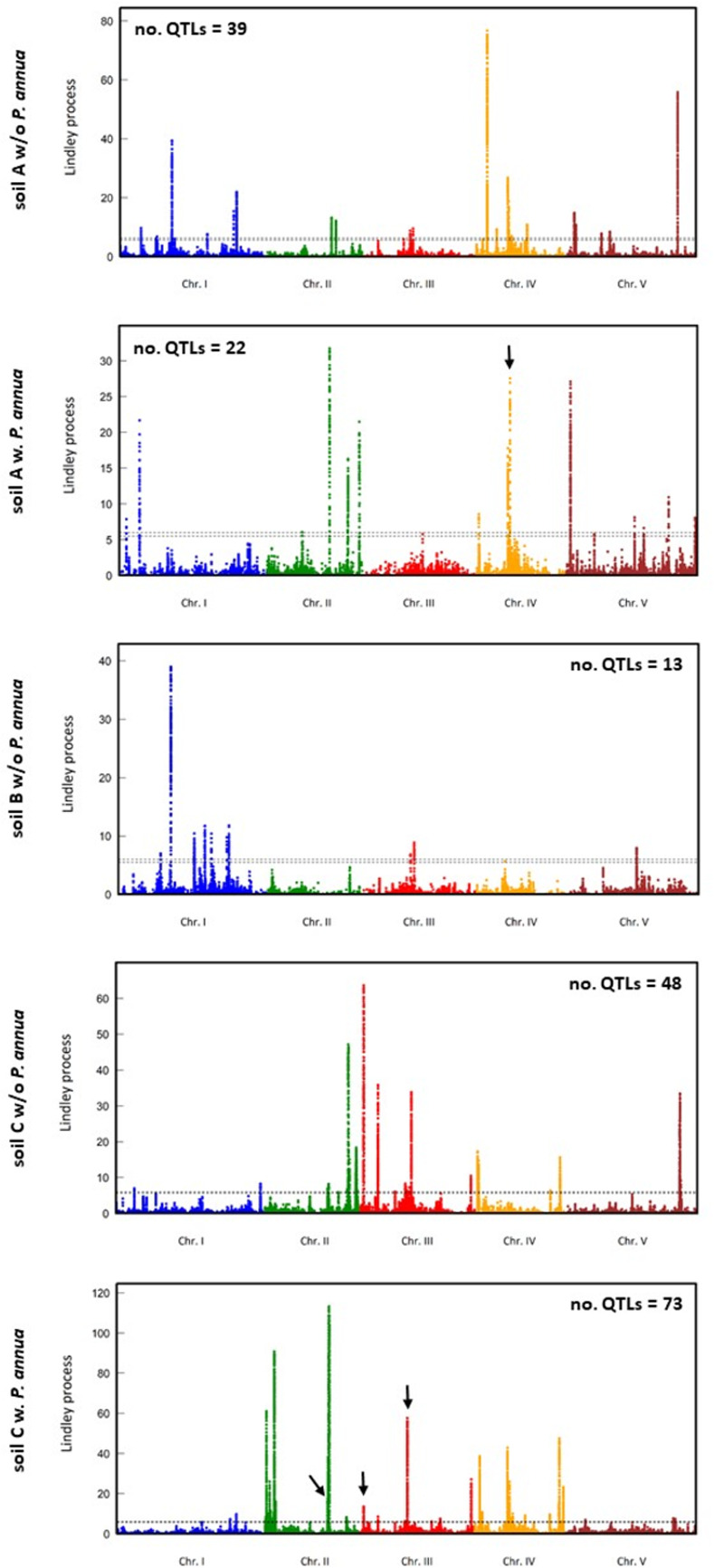
A polygenic architecture underlying natural genetic variation in disease index within each micro-habitat for which disease index was significantly heritable. Manhattan plot of the Lindley process (local score method with a tuning parameter ξ = 2). The x-axis indicates the physical position of the 981,617 SNPs with a minor allele relative frequency above 7%, along the five chromosomes. The dashed lines indicate the minimum and maximum of the five chromosome-wide significance thresholds. Black arrows indicate the positions of the four QTLs containing candidate genes common between disease index and total seed production (see Fig 6).

**Fig 5:**
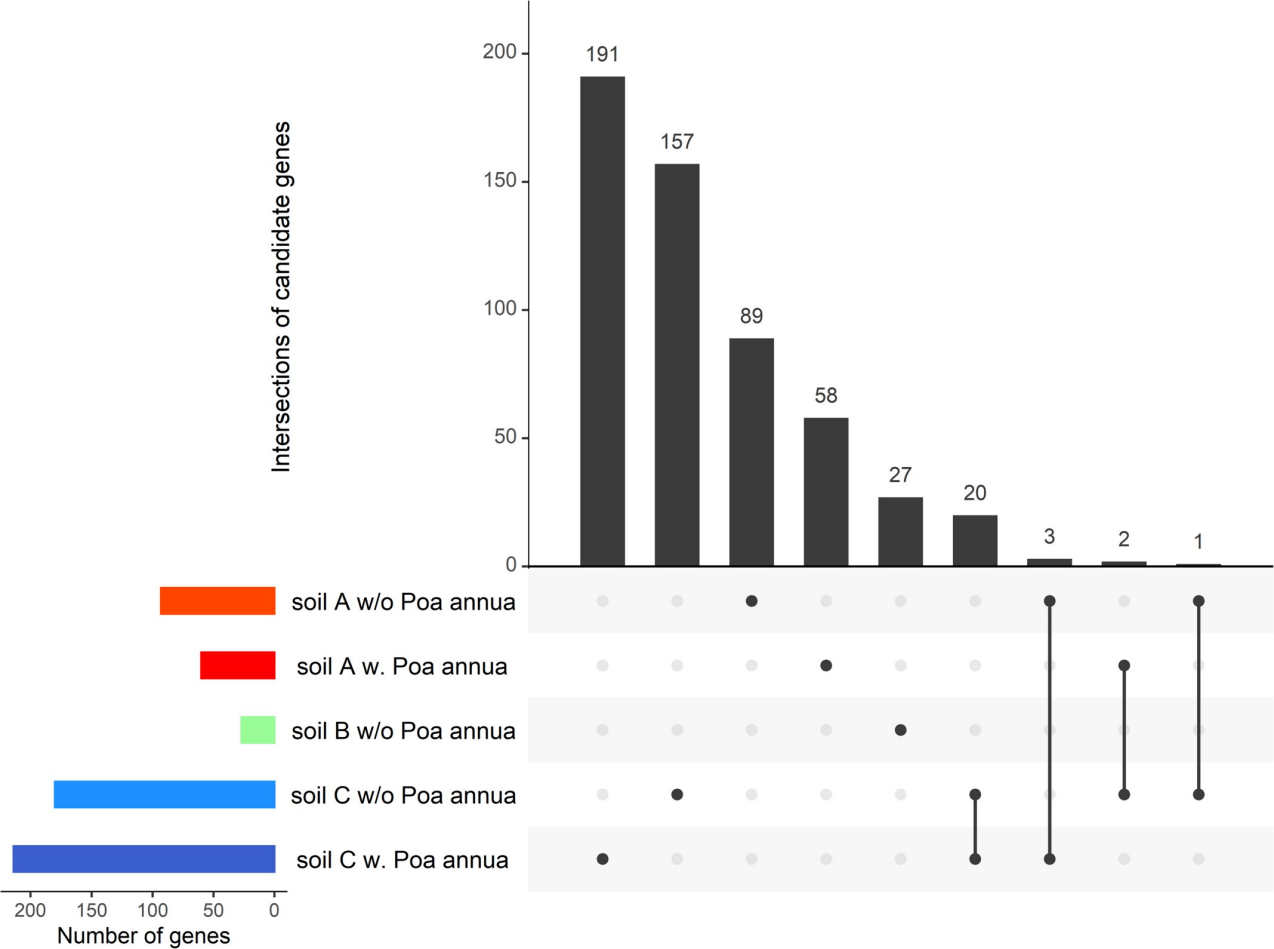
A variable architecture underlying natural genetic variation in disease index within each micro-habitat for which disease index was significantly heritable. UpSet plot illustrating the number of candidate genes that were either specific to a single micro-habitat (i.e. single black dots) or common between two micro-habitats (i.e. black dots connected by a solid line). ‘w/o’: absence of *P*. *annua*, ‘w.’: presence of *P*. *annua*. For each micro-habitat, the number of candidate genes identified by GWA mapping (colored bars) corresponds to the sum of the numbers of candidate genes above the grey bars for which dots are present. For instance, the total number of candidate genes identified by GWA mapping for the micro-habitat ‘soil C w. *Poa annua*’ is 214, which corresponds to the sum of the values 191, 20 and 3.

Natural genetic variation for disease index was highly polygenic, with the detection of between 13 and 73 QTLs depending on microhabitat (Fig 4), for a total of 195 detected QTLs overlapping with 548 unique candidate genes (Dataset 3 in S1 Dataset). The genetic architecture was highly variable among the micro-habitats, with most candidate genes being specific to a particular micro-habitat (Fig 5). The main exception concerns 20 candidate genes that were common both in absence and in presence of *P*. *annua* in soil C (Fig 5). Note that no candidate gene associated with disease index was detected as common between more than two micro-habitats (Fig 5). A similar pattern was observed for natural genetic variation of fecundity (S4 Fig), with the detection of between 14 to 32 QTLs depending on micro-habitat. The 107 detected QTLs overlaps with 360 candidate genes (Dataset 3 in S1 Dataset), most of them being specific to a particular micro-habitat (S4 Fig).

**Fig 6:**
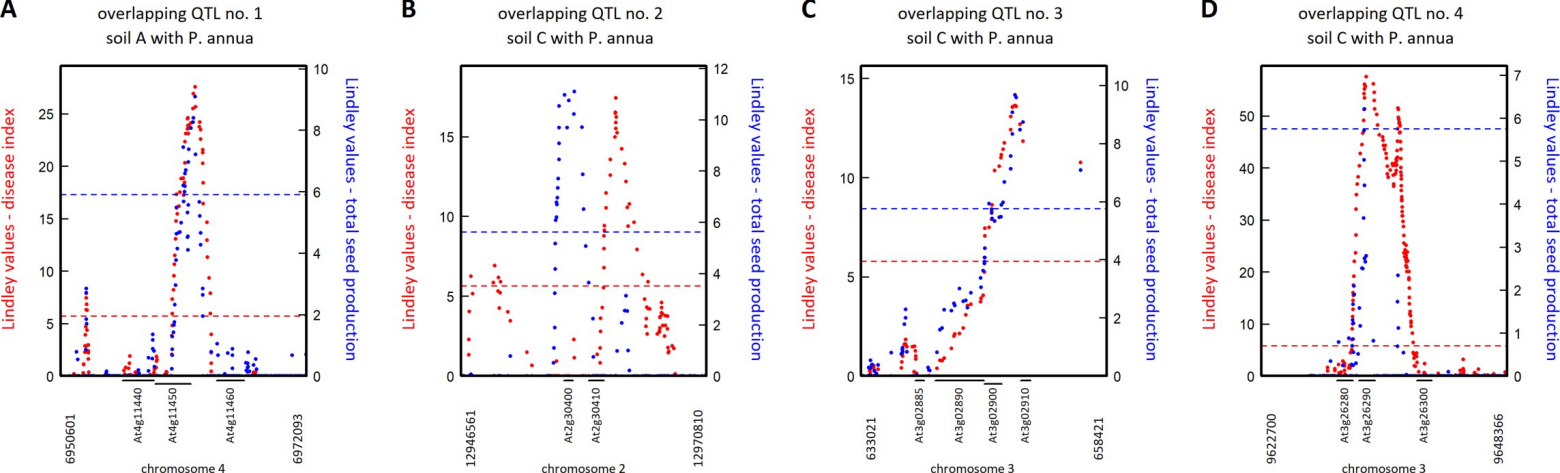
Zoom spanning the four genomic regions containing candidate genes common between disease index and total seed production. (A) Overlapping QTL between disease index and total seed production on soil A in presence of *P*. *annua*. (B-D) Overlapping QTLs between disease index and total seed production on soil C in presence of *P*. *annua*. Red and blue dots correspond to Lindley values for disease index and total seed production, respectively. The red and blue dashed lines indicate the corresponding chromosome-wide significance threshold for disease index and total seed production, respectively. Vertical numbers (expressed in bp) at the bottom of each panel correspond to the physical positions of the QTL region.

Only four QTLs were common between disease index and fecundity, suggesting that only a small fraction of disease resistance QTLs detected in the local mapping population TOU-A are under selection in the micro-habitats tested in this study (Figs 4 and S6). One QTL was detected in soil A in presence of *P*. *annua* and three QTLs were detected in soil C in presence of *P*. *annua* (Fig 4). The first QTL is located between the genes *AT4G11450* and *AT4G11460* encoding a protein of unknown function and the cysteine-rich receptor-like protein kinase CRK30, respectively (Fig 6). The second QTL is located on the genes *OVATE FAMILY PROTEIN 2* (*OFP2*) and *TUBULIN FOLDING FACTOR A* (also named *KIS*) (Fig 6). The third QTL is located on genes *AT3G02900* and *AT3G02910* encoding a receptor-like protein and an AIG2-like (avirulence induced gene) family protein, respectively (Fig 6). The last QTL is located on the gene *AT3G26290* encoding the cytochrome P450 protein CYP71B26 (Fig 6).

## Discussion

### Extensive Genotype × Environment interactions should promote the maintenance of quantitative disease resistance polymorphisms in the local TOU-A population

In this study, we detected extensive genetic variation for quantitative disease resistance as well as extensive GxE interactions at a very small spatial scale. In previous experiments conducted under laboratory controlled conditions, extensive genetic variation was detected in the TOU-A population for either qualitative or quantitative disease resistance, when accessions were mono-infected with strains of the bacterial pathogens *Pseudomonas syringae*, *R*. *solanacearum* and *X*. *campestris* [24, 40, 48, 49]. However, coinfection with multiple pathogens or multiple isolates from the same pathogenic species appears the norm in natural populations of *A*. *thaliana* [1, 12, 27, 50]. Whether plants grown in the six micro-habitats were attacked by either a single pathogenic isolate, multiple pathogenic isolates or multiple pathogens remains an open question and would have required the characterization of the entire pathobiota (i.e. viruses, bacteria, fungi and oomycetes) using, for instance, a shotgun metagenomics approach [51].

The environmental heterogeneity encountered by the accessions of *A*. *thaliana* in the TOU-A population affected in an unpredictable way the mean level of disease resistance as well as the extent of genetic variation, suggesting non-linear interacting effects between soil agronomic properties and heterospecific competition on plant-pathogen interactions. We may caution that differences in soil agronomic properties can lead to differences in soil microbial communities that directly mediates plant-pathogen interactions, as previously demonstrated in the natural plant pathosystems *Plantago lanceolata—Podosphaera plantaginis* [20]. Based on a correlation approach, pH was proposed as one of the main factors shaping the microbiota (bacteria, fungi and oomycetes) of *A*. *thaliana* across 17 natural European sites, in the soil, rhizosphere, rhizoplane and root compartments [29]. Because pH varies between 4.8 and 6.2 along the 350-m transect of the TOU-A population, it would be interesting to characterize in each micro-habitat, microbial communities of the soil and in diverse plant compartments.

Importantly, although accessions switched ranks for disease resistance among the micro-habitats, the fecundity-disease resistance relationship was consistently negative, which is line with the negative relationship detected between disease severity and total seed production in the *A*. *thaliana–P*. *syringae* pathosystem under growth chamber conditions [52]. In combination with the spatial grain of genetic variation in disease resistance that differs among the micro-habitats, these results suggest the presence of heterogeneous selection acting on disease resistance in the TOU-A population from the scale of few meters to the scale of several tens of meters. With alternative host genotypes being favored under contrasting environmental conditions, such a heterogeneous selection should promote the maintenance of polymorphism at the resistance genes. This is in line with the presence of long-lived haplotypes in the TOU-A population for the *R* genes *RPM1* and *RPS2* conferring qualitative resistance against *P*. *syringae* strains, and the gene *RKS1* conferring broad-spectrum quantitative resistance against *X*. *campestris* [24, 49]. The presence of long-lived haplotypes at *R* genes and *RKS1* was also observed in several tens of populations located in Europe [40, 49, 53]. Therefore, environmentally driven heterogeneous selection may be widespread across the native range of *A*. *thaliana*, in particular in populations inhabiting environments with contrasting soil agronomic properties and/or containing a large number of companion plant species [26, 33].

### Why so few common QTLs between disease resistance and total seed production?

A polygenic and habitat-dependent genetic architecture was detected for both disease resistance and fecundity. Similar patterns were observed (i) under laboratory controlled conditions when the same set of 195 TOU-A accessions were challenged with the bacterial pathogen *R*. *solanacearum* at two temperatures differing by only 3°C [48], and (ii) under field conditions when a set of worldwide accessions of *A*. *thaliana* were phenotyped for fecundity in four sites across Europe [54].

However, despite a significant negative genetic relationship between disease resistance and total seed production, the number of common QTLs between these two traits was small. Several non-exclusive hypotheses can be advanced to explain the paucity of QTLs overlapping between disease resistance and total seed production. Firstly, we scored disease symptoms in a semi-quantitative manner that may have led to a subjective categorization of some plants according to the scale of scoring. Although substantial genetic variation was detected among the 195 local accessions for disease resistance, some mis-categorization may in turn have affected the estimates of genotypic values, and thus an accurate detection of the QTLs associated with disease resistance. Secondly, a non-negligible fraction of disease resistance variation can be neutral, in particular in micro-habitats where total seed production is weakly associated with disease index, such as in soil A in presence of *P*. *annua*. Thirdly, total seed production is only one component of fitness measured in this study. It would be informative to consider other fitness components, such as seed quality, germination rate and survival rate of offspring [55]. Fourthly, despite the detection of dozens of QTLs for each trait in each micro-habitat, the number of accessions phenotyped in this study might have been too small to correctly characterize the genetic architecture of such integrative traits like quantitative disease resistance and total seed production, thereby impeding the detection of QTLs with very small effects [54, 56]. Fifthly, as proposed in [52], infection might have affected total seed production through different genetic mechanisms than the ones associated with disease resistance in this study, such as disease tolerance. In line with disease tolerance, the fraction of the pathobiota in the leaf bacterial microbiota was high in a non-negligible number of symptomless *A*. *thaliana* plants collected in natural populations located south-west of France, suggesting the presence in these plants of genetic mechanisms allowing pathogen population growth without affecting plant development [27]. Sixthly, selection differentials include both direct selection on a trait and indirect selection due to selection acting on correlated traits [44]. Therefore, considering additional putative adaptive traits such as the duration of reproductive period or the number of branches [26, 57], may help to measure the strength and trend of directional selection acting on disease resistance through the estimation of linear partial regression coefficients [58]. Combined with pairwise genetic correlations, a multivariate genotypic selection analysis may in turn forecast genetic constraints between disease resistance and other phenotypic traits [58].

### Candidate genes for disease resistance potentially under selection encode diverse molecular functions

The candidate genes associated with natural variation of quantitative disease resistance and potentially under selection encode diverse molecular functions, suggesting that the molecular mechanisms underlying this type of resistance may be more complex than anticipated [59]. Such a pattern is in agreement with previous studies conducted on *A*. *thaliana* when challenged with diverse pathogens including viruses [35], bacteria [6, 40, 48, 49, 60, 61], fungi [38, 39, 62] and oomycete [63, 64].

In this study, two candidate genes are of particular interest. Firstly, the cysteine-rich receptor-like protein kinase CRK30 is a member of one of the largest group of receptor-like kinases in plants [65]. The transcript level of several CRKs has been reported to be induced by bacterial pathogens [65]. Recently, by assessing transcriptional response of *A*. *thaliana* to 39 commensal bacterial strains in the leaf compartment, a core set of 24 genes (including two CRKs) consistently induced by the presence of most strains, was identified and referred as a molecular process called general non-self-response (GNSR) [66]. These findings suggest that in our study, CRK30 may have directly perceived bacterial pathogens and/or indirectly perceived microbiota perturbations by pathogen invasion. Secondly, under controlled laboratory conditions, the transcript level of the cytochrome P450 gene *CYP71B26* was deregulated in the early stages of infection with *X*. *campestris* [59], which is the most prevalent and abundant bacterial pathogen in natural populations of *A*. *thaliana* located south-west of France [12, 27]. For the five other candidate genes associated with both disease index and fecundity, no obvious links between the function of these genes and disease index was reported in the literature.

The next step to understanding the mechanisms underlying natural variation of quantitative disease resistance in ecologically relevant habitats would be to functionally validate the two candidate genes related to response to bacterial pathogens, i.e. *CRK30* and *CYP71B26*, and test their effects on total seed production. By growing isogenic lines differing only by natural alleles present in the TOU-A population at these two genes on the six micro-habitats, it may help to better understand at the genetic level the adaptive dynamics of host-pathobiota interactions in a spatially fine-grained environment [67].

## Methods

### Plant material and experimental design

One hundred and ninety-five accessions of *A*. *thaliana* of the local TOU-A population were used in this study. This set of accessions were collected in 2002 (n = 80) and 2010 (n = 115) according to a sampling scheme based on the density of *A*. *thaliana* plants along a 350-m transect under an electric fence separating two permanent meadows. We reduced differences in maternal effects among the 195 accessions by growing one plant per accession for one generation, under greenhouse conditions (16-h photoperiod, 20°C).

The experimental design was fully described in Frachon *et al*. [24]. Briefly, the 195 accessions were grown in six ‘soil × interspecific competition’ micro-habitats at the local site of the TOU-A population. Each was organized in five blocks. Each block corresponded to an independent randomization of 195 plants with one replicate per accession, for a total of 5,850 plants across the six micro-habitats.

Disease symptoms were scored on March 5^th^, 2013. Maximal rosette diameter (to the nearest millimeter) used in this study was measured before the onset of winter on November 21^st^, 2012 and used as a proxy for plant size. Bolting was scored on March 5^th^, 2013 as the presence of an inflorescence distinguishable from the leaves at a size > 5 mm. Total seed production was previously estimated by multiplying the number of fertilized fruits by an estimate of their corresponding fruit length [24].

### Natural genetic variation

Natural variation of disease index was analysed using the following statistical mixed model:

$${\text{diseases}\;\text{index}}_{\text{ijklmn}}\text{=}\mu_{\text{disease}\;\text{index}}+{\text{block}}_{\text{i}}\left({\text{soil}}_{\text{j}}\times {\text{comp}}_{\text{k}}\right)+{\text{soil}}_{\text{j}}+{\text{comp}}_{\text{k}}+{\text{soil}}_{\text{j}}\times {\text{comp}}_{\text{k}}+{\text{acc}}_{\text{l}}+{\text{acc}}_{\text{l}}\times {\text{soil}}_{\text{j}}+{\text{accl}\times \text{comp}}_{\text{k}}+{\text{acc}}_{\text{l}}\times {\text{soil}}_{\text{j}}\times {\text{comp}}_{\text{k}}+{\text{bolting}}_{\text{m}}\left({\text{soil}}_{\text{j}}\times {\text{comp}}_{\text{k}}\right)+{\text{diameter}}_{\text{n}}\left({\text{soil}}_{\text{j}}\times {\text{comp}}_{\text{k}}\right)+\varepsilon_{\text{ijklmn}}$$ (Model 1)

In this model, *μ* is the overall phenotypic mean; ‘block’ accounts for differences between the five experimental blocks within each type of ‘soil × absence or presence of *P*. *annua*’ experimental combination; ‘soil’ corresponds to the effects of the three types of soil; ‘comp’ measures the effect of the presence of *P*. *annua*; ‘acc’ measures the differences among the195 accessions; interaction terms involving the accession term account for genetic variation in reaction norms of accessions between the three types of soil and the absence or presence of *P*. *annua*; ‘bolting’ measures the effect of being bolted on the day plants were scored for disease symptoms; ‘diameter’ accounts for developmental effects approximated by maximal rosette dimeter before the onset of winter; and ε is the residual term. Inference was performed using ReML estimation, using the PROC MIXED procedure in SAS v.9.4 (SAS Institute Inc., Cary, North Carolina, USA). All factors were treated as fixed effects, except the term ‘acc’, which was treated as a random effect. For fixed effects, terms were tested over their appropriate denominators for calculating *F* values. Significance of the random effects was determined by likelihood ratio tests of the model with and without these effects.

Best linear unbiased estimates (BLUEs) of disease index were obtained for each accession in each of the six micro-habitats by running the following mixed model (PROC MIXED procedure in SAS v.9.4):

$${\text{diseases}\;\text{index}}_{\text{ilmn}}\text{=}\mu +{\text{block}}_{\text{i}}+{\text{acc}}_{\text{l}}+{\text{bolting}}_{\text{m}}+{\text{diameter}}_{\text{n}}+\varepsilon_{\text{ilmn}}$$ (Model 2)

Because *A*. *thaliana* is a highly selfing species [23], BLUEs correspond to the genotypic values of accessions. Based on the same individual plants scored for disease index, BLUEs for fecundity were obtained from Frachon *et al*. [24].

Broad-sense heritability (*H*^2^) of disease index was estimated from variance component estimates for the ‘block’ and ‘acc’ terms (PROC VARCOMP procedure in SAS v.9.4) on the residuals obtained after fitting Model (2) without the ‘block’ and ‘acc’ terms.

### Spatial grains of disease index variation

For each micro-habitat, the spatial grain of disease index variation was estimated by first modeling a spectral decomposition of the spatial relationships among the 195 accessions with PCNMs, using the *pcnm* function implemented in the *R vegan* package [68] using the Euclidean distance matrix based on the coordinates of the 195 accessions along the 350-m transect. The resulting orthogonal PCNM components correspond to successive spatial grains [69]. The first and last PCNM components define large and fine spatial grains, respectively [69]. Then, for each micro-habitat, all PCNM components were used as explanatory variables in a multiple linear regression on genetic variation in disease index in the *R* environment. Multiple testing were controlled for a false discovery rate (FDR) at a nominal level of 5% [70].

### Genotypic selection analysis

The extent of natural selection on disease resistance was measured by the selection differential S [43, 44], within each of the five micro-habitats for which disease index was significantly heritable. Based on BLUEs, we first calculated in each micro-habitat relative fecundity as the fecundity estimate divided by the mean fecundity estimate, and disease index standardized to a mean of zero and a standard deviation of one [58]. The following analysis of covariance (ANCOVA) was then run in each micro-habitat (PROC GLM procedure in SAS v.9.4), with the S estimate corresponding to the regression slope value:

$${\text{relative}\;\text{fitness}}_{\text{d}}\text{=}\mu_{\text{relative}\;\text{fitness}}+{\text{disease}\;\text{index}}_{\text{d}}+\varepsilon_{\text{d}}$$ (Model 3)

To test whether the fecundity-disease index relationship was affected by the soil type and/or the presence/absence of *P*. *annua*, we run the following ANCOVA (PROC GLM procedure in SAS v.9.4):

$${\text{relative}\;\text{fitness}}_{\text{djkl}}\text{=}\mu_{\text{relative}\;\text{fitness}}+{\text{disease}\;\text{index}}_{\text{d}}+{\text{soil}}_{\text{j}}+{\text{comp}}_{\text{k}}+{\text{soil}}_{\text{j}}\times {\text{comp}}_{\text{k}}+{\text{disease}\;\text{index}}_{\text{d}}\times {\text{soil}}_{\text{j}}+{\text{disease}\;\text{index}}_{\text{d}}\times {\text{comp}}_{\text{k}}+{\text{disease}\;\text{index}}_{\text{d}}\times {\text{soil}}_{\text{j}}\times {\text{comp}}_{\text{k}}+\varepsilon_{\text{djklmn}}$$ (Model 4)

In Models (3) and (4), *μ* is the overall phenotypic mean; ‘disease index’ corresponds to disease index standardized within each micro-habitat; ‘soil’ corresponds to the effects of the three types of soil; ‘comp’ measures the effect of the presence of *P*. *annua*; and ε is the residual term.

### Genome-Wide Association mapping combined with a local score approach

Although the effects of population structure on phenotype-SNP associations were demonstrated to be limited in the TOU-A population [26, 32], we nonetheless run GWA mapping using a mixed model implemented in the software EMMAX (Efficient Mixed-Model Association eXpedited) [71]. To control for the effect of population structure, we included as a covariate an identity-by-state kinship matrix *K*. This kinship matrix was based on 1,902,592 SNPs identified among the 195 accessions of the TOU-A population [24]. Because rare alleles may increase the rate of false positives [63, 72], we only considered SNPs with a minor allele relative frequency (MARF) > 7%, a MARF value above which the *p* value distribution obtained from the EMMAX mixed model is not dependent on MARF values in the TOU-A population [24]. Based on the resulting 981,617 SNPs, the EMMAX mixed model was run on BLUEs of disease index. Results from EMMAX mixed model based on BLUEs for fecundity were previously obtained in Frachon *et al*. [24].

In order to better characterize the genetic architecture associated with natural genetic variation in disease index and fecundity, we applied a local score approach on the set of *p*-values provided by EMMAX. This local score approach increases the power of detecting QTLs with small effect and narrows the size of QTL genomic regions [47, 73]. A tuning parameter ξ of 2 expressed in–log_10_ scale, was used in this study. Significant phenotype-SNP associations were identified by estimating a chromosome-wide significance threshold for each chromosome [47]. Because the estimation of the significance threshold depends on the distribution of the *p*-values after a FDR control, threshold values depend on both the identity of the phenotypic trait and the chromosome identity. Based on a custom script [74], we retrieved all candidate genes underlying QTLs by selecting all genes inside the QTL regions as well as the first gene upstream and the first gene downstream of these QTL regions (Dataset 3 in S1 Dataset). The TAIR 10 database (https://www.arabidopsis.org/) was used as our reference. The number of candidate genes that were either specific to a single micro-habitat or common between several micro-habitats were illustrated by UpSet plots using the package UpSetR implemented under the *R* environment [75].

## Supporting information

S1 Dataset This file includes the three data sets mentioned in the main text. (XLSX)

S1 Table Genetic variation among the 195 TOU-A accessions for disease index within each six micro-habitat. The random effect ‘Accession’ is in italic. The model random term was tested with likelihood ratio tests of model with and without this effect. Bold *P*-values indicate significant effect after Bonferroni correction. LRT: Likelihood Ratio Test. ‘w/o’: absence of *P*. *annua*, ‘w.’: presence of *P*. *annua*.
(DOCX)

S2 Table Spatial genetic variation of disease index. Levels of significance (*p*-values) between disease index variation and each Principal Coordinates of Neighbor Matrices (PCNM) component within each micro-habitat for which disease index was significantly heritable. Significant associations after a FDR adjustment at a nominal level of 5% are highlighted in green.
(DOCX)

S3 Table Genotypic selection analysis revealing fecundity–disease index relationship. Bold *P*-values indicate significant effect after Bonferroni correction. ‘soil’: soils A, B and C. ‘comp’: absence or presence of *P*. *annua*.
(DOCX)

S1 Fig Variation of 14 edaphic factors along the 350-m transect in the TOU-A population illustrated by Jitter plots to better visualize overlapping individual one-dimensional values. Each dot corresponds to one of the 83 soil samples collected along the 350-m transect and characterized for 14 edaphic factors; i.e. pH, maximal water holding capacity (WHC), total nitrogen content (N), organic carbon content (C), C/N ratio, soil organic matter content (SOM), concentrations of P_2_O_5_, K, Ca, Mg, Mn, Al, Na and Fe. Red, green and blue dots correspond to soil samples located in the three contrasted natural soils on which plants were grown in this study, i.e. A (n = 3), B (n = 9) and C (n = 8), respectively. The remaining grey dots (n = 63) correspond to soil samples located outside of the three contrasted natural soils tested in this study.
(DOCX)

S2 Fig Relationship between disease index and maximal rosette diameter within each micro-habitat. A w/o Poa’: soil A in absence of *Poa annua*, ‘A w. Poa’: soil A in presence *Poa annua*, ‘B w/o Poa’: soil B in absence of *Poa annua*, ‘B w. Poa’: soil B in presence *Poa annua*, ‘C w/o Poa’: soil C in absence of *Poa annua*, ‘C w. Poa’: soil C in presence *Poa annua*. The solid line corresponds to the fitted regression line, whereas the dashed lines delimit the band of 99% confidence intervals. ‘R²’ corresponds to the adjusted R-squared of the fitted model. *** *P* < 0.001.
(DOCX)

S3 Fig Spectral decomposition of the relationships among the 195 accessions along the 350-m transect. The 59 Principal Coordinates of Neighbor Matrices (PCNM) components are ranking from the higher (PCNM1) to the lower (PCNM59) eigenvalues *i*.*e*. from coarse-grained to finer-grained spatial variations. The size of squares are proportional to the PCNM values. The filled and open squares indicate negative and positive PCNM values, respectively. The x-axis corresponds to the length of the transect along which the 195 TOU-A accessions have been collected.
(DOCX)

S4 Fig A polygenic architecture underlying natural genetic variation of total seed production within each micro-habitat for which disease index was significantly heritable. Manhattan plot of the Lindley process (local score method with a tuning parameter ξ = 2). The x-axis indicates the physical position of the 981,617 SNPs along the five chromosomes. The dashed lines indicate the minimum and maximum of the five chromosome-wide significance thresholds.
(DOCX)

S5 Fig A variable architecture underlying natural genetic variation of total seed production within each micro-habitat for which disease index was significantly heritable. UpSet plot illustrating the number of candidate genes that were either specific to a single micro-habitat (i.e. single black dots) or common between two micro-habitats (i.e. black dots connected by a solid line). ‘w/o’: absence of *P*. *annua*, ‘w.’: presence of *P*. *annua*. For each micro-habitat, the number of candidate genes identified by GWA mapping (colored bars) corresponds to the sum of the numbers of candidate genes above the grey bars for which dots are present. For instance, the total number of candidate genes identified by GWA mapping for the micro-habitat ‘soil C w:O *Poa annua*’ is 55, which corresponds to the sum of the values 51, 3 and 1.
(DOCX)

S6 Fig Comparison of the polygenic architecture underlying natural genetic variation of disease index and total seed production within each micro-habitat. (A) soil A in absence of *Poa annua*. (B) soil A in presence *Poa annua*. (C) soil B in absence of *Poa annua*. (D) soil C in absence of *Poa annua*. (E) soil C in presence *Poa annua*. Manhattan plot of the Lindley process (local score method with a tuning parameter ξ = 2). The x-axis indicates the physical position of the 981,617 SNPs along the five chromosomes. The dashed lines indicate the minimum and maximum of the five chromosome-wide significance thresholds.
(DOCX)
