# Supplementary material for: A Genome-Wide Association study in Arabidopsis thaliana to decipher the adaptive genetics of quantitative disease resistance in a native heterogeneous environment
Source: PLoS One. 2022 Oct 3;17(10):e0274561. doi: 10.1371/journal.pone.0274561 (PMC9529085; doi:10.1371/journal.pone.0274561)
Supplement: S1 Table — The random effect ‘Accession’ is in italic. The model random term was tested with likelihood ratio tests of model with and without this effect. Bold P-values indicate significant effect after Bonferroni correction. LRT: Likelihood Ratio Test. ‘w/o’: absence of P. annua, ‘w.’: presence of P. annua. (DOCX) [file pone.0274561.s002.docx]

**S1 Table.** **Genetic variation among the 195 TOU-A accessions for disease index within each six micro-habitat.** The random effect ‘Accession’ is in italic. The model random term was tested with likelihood ratio tests of model with and without this effect. Bold *P*-values indicate significant effect after Bonferroni correction. LRT: Likelihood Ratio Test. ‘w/o’: absence of *P. annua*, ‘w.’: presence of *P. annua*.
