# Supplementary material for: A Genome-Wide Association study in Arabidopsis thaliana to decipher the adaptive genetics of quantitative disease resistance in a native heterogeneous environment
Source: PLoS One. 2022 Oct 3;17(10):e0274561. doi: 10.1371/journal.pone.0274561 (PMC9529085; doi:10.1371/journal.pone.0274561)
Supplement: S2 Table — Levels of significance (p-values) between disease index variation and each Principal Coordinates of Neighbor Matrices (PCNM) component within each micro-habitat for which disease index was significantly heritable. Significant associations after a FDR adjustment at a nominal level of 5% are highlighted in green. (DOCX) [file pone.0274561.s003.docx]

**S2 Table. Spatial genetic variation of disease index. Levels of significance (*p*-values) between disease index variation and each Principal Coordinates of Neighbor Matrices (PCNM) component within each micro-habitat for which disease index was significantly heritable.** Significant associations after a FDR adjustment at a nominal level of 5% are highlighted in green.
