# Supplementary material for: A Genome-Wide Association study in Arabidopsis thaliana to decipher the adaptive genetics of quantitative disease resistance in a native heterogeneous environment
Source: PLoS One. 2022 Oct 3;17(10):e0274561. doi: 10.1371/journal.pone.0274561 (PMC9529085; doi:10.1371/journal.pone.0274561)
Supplement: S3 Table — Bold P-values indicate significant effect after Bonferroni correction. ‘soil’: soils A, B and C. ‘comp’: absence or presence of P. annua. (DOCX) [file pone.0274561.s004.docx]

**S3 Table. Genotypic selection analysis revealing fecundity – disease index relationship**. Bold *P*-values indicate significant effect after Bonferroni correction. ‘soil’: soils A, B and C. ‘comp’: absence or presence of *P. annua*.
