# Supplementary material for: A Genome-Wide Association study in Arabidopsis thaliana to decipher the adaptive genetics of quantitative disease resistance in a native heterogeneous environment
Source: PLoS One. 2022 Oct 3;17(10):e0274561. doi: 10.1371/journal.pone.0274561 (PMC9529085; doi:10.1371/journal.pone.0274561)

**S1 Figure. Variation of 14 edaphic factors along the 350-m transect in the TOU-A population illustrated by Jitter plots to better visualize overlapping individual one-dimensional values.** Each dot corresponds to one of the 83 soil samples collected along the 350-m transect and characterized for 14 edaphic factors; i.e. pH, maximal water holding capacity (WHC), total nitrogen content (N), organic carbon content (C), C/N ratio, soil organic matter content (SOM), concentrations of P_2_O_5_, K, Ca, Mg, Mn, Al, Na and Fe. Red, green and blue dots correspond to soil samples located in the three contrasted natural soils on which plants were grown in this study, i.e. A (n = 3), B (n = 9) and C (n = 8), respectively. The remaining grey dots (n = 63) correspond to soil samples located outside of the three contrasted natural soils tested in this study.


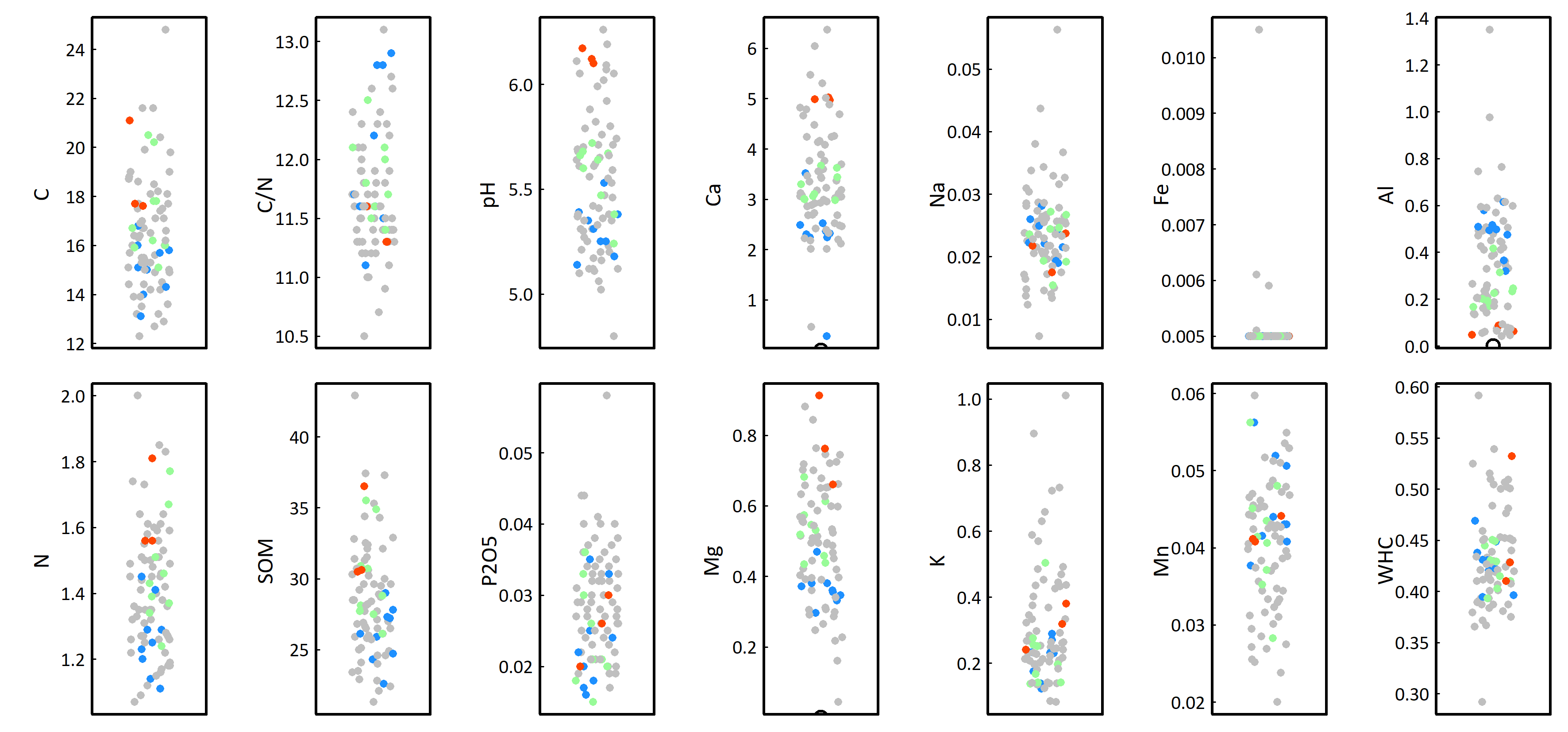

Supplement: S1 Fig — Each dot corresponds to one of the 83 soil samples collected along the 350-m transect and characterized for 14 edaphic factors; i.e. pH, maximal water holding capacity (WHC), total nitrogen content (N), organic carbon content (C), C/N ratio, soil organic matter content (SOM), concentrations of P2O5, K, Ca, Mg, Mn, Al, Na and Fe. Red, green and blue dots correspond to soil samples located in the three contrasted natural soils on which plants were grown in this study, i.e. A (n = 3), B (n = 9) and C (n = 8), respectively. The remaining grey dots (n = 63) correspond to soil samples located outside of the three contrasted natural soils tested in this study. (DOCX) [file pone.0274561.s005.docx]
