# Supplementary material for: A Genome-Wide Association study in Arabidopsis thaliana to decipher the adaptive genetics of quantitative disease resistance in a native heterogeneous environment
Source: PLoS One. 2022 Oct 3;17(10):e0274561. doi: 10.1371/journal.pone.0274561 (PMC9529085; doi:10.1371/journal.pone.0274561)
Supplement: S2 Fig — A w/o Poa’: soil A in absence of Poa annua, ‘A w. Poa’: soil A in presence Poa annua, ‘B w/o Poa’: soil B in absence of Poa annua, ‘B w. Poa’: soil B in presence Poa annua, ‘C w/o Poa’: soil C in absence of Poa annua, ‘C w. Poa’: soil C in presence Poa annua. The solid line corresponds to the fitted regression line, whereas the dashed lines delimit the band of 99% confidence intervals. ‘R²’ corresponds to the adjusted R-squared of the fitted model. *** P < 0.001. (DOCX) [file pone.0274561.s006.docx]

**S2 Figure. Relationship between disease index and maximal rosette diameter within each micro-habitat**. A w/o Poa’ : soil A in absence of *Poa annua*, ‘A w. Poa’ : soil A in presence *Poa annua*, ‘B w/o Poa’ : soil B in absence of *Poa annua*, ‘B w. Poa’ : soil B in presence *Poa annua*, ‘C w/o Poa’ : soil C in absence of *Poa annua*, ‘C w. Poa’ : soil C in presence *Poa annua*. The solid line corresponds to the fitted regression line, whereas the dashed lines delimit the band of 99% confidence intervals. ‘R²’ corresponds to the adjusted R-squared of the fitted model. *** *P* < 0.001.

**
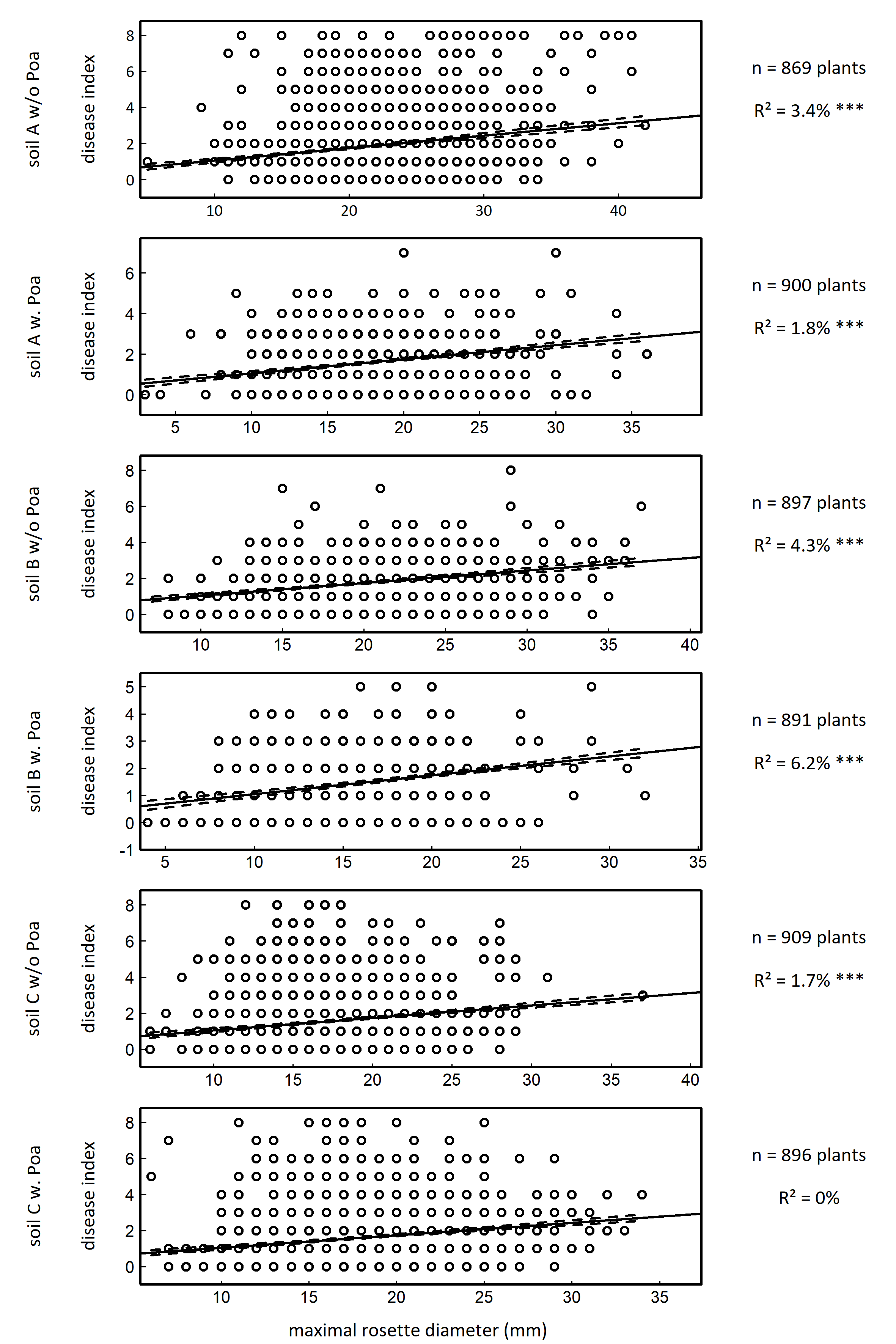
**
