# Supplementary material for: A Genome-Wide Association study in Arabidopsis thaliana to decipher the adaptive genetics of quantitative disease resistance in a native heterogeneous environment
Source: PLoS One. 2022 Oct 3;17(10):e0274561. doi: 10.1371/journal.pone.0274561 (PMC9529085; doi:10.1371/journal.pone.0274561)
Supplement: S3 Fig — The 59 Principal Coordinates of Neighbor Matrices (PCNM) components are ranking from the higher (PCNM1) to the lower (PCNM59) eigenvalues i.e. from coarse-grained to finer-grained spatial variations. The size of squares are proportional to the PCNM values. The filled and open squares indicate negative and positive PCNM values, respectively. The x-axis corresponds to the length of the transect along which the 195 TOU-A accessions have been collected. (DOCX) [file pone.0274561.s007.docx]

**S3 Figure. Spectral decomposition of the relationships among the 195 accessions along the 350-m transect.** The 59 Principal Coordinates of Neighbor Matrices (PCNM) components are ranking from the higher (PCNM1) to the lower (PCNM59) eigenvalues *i.e.* from coarse-grained to finer-grained spatial variations. The size of squares are proportional to the PCNM values. The filled and open squares indicate negative and positive PCNM values, respectively. The x-axis corresponds to the length of the transect along which the 195 TOU-A accessions have been collected.

**
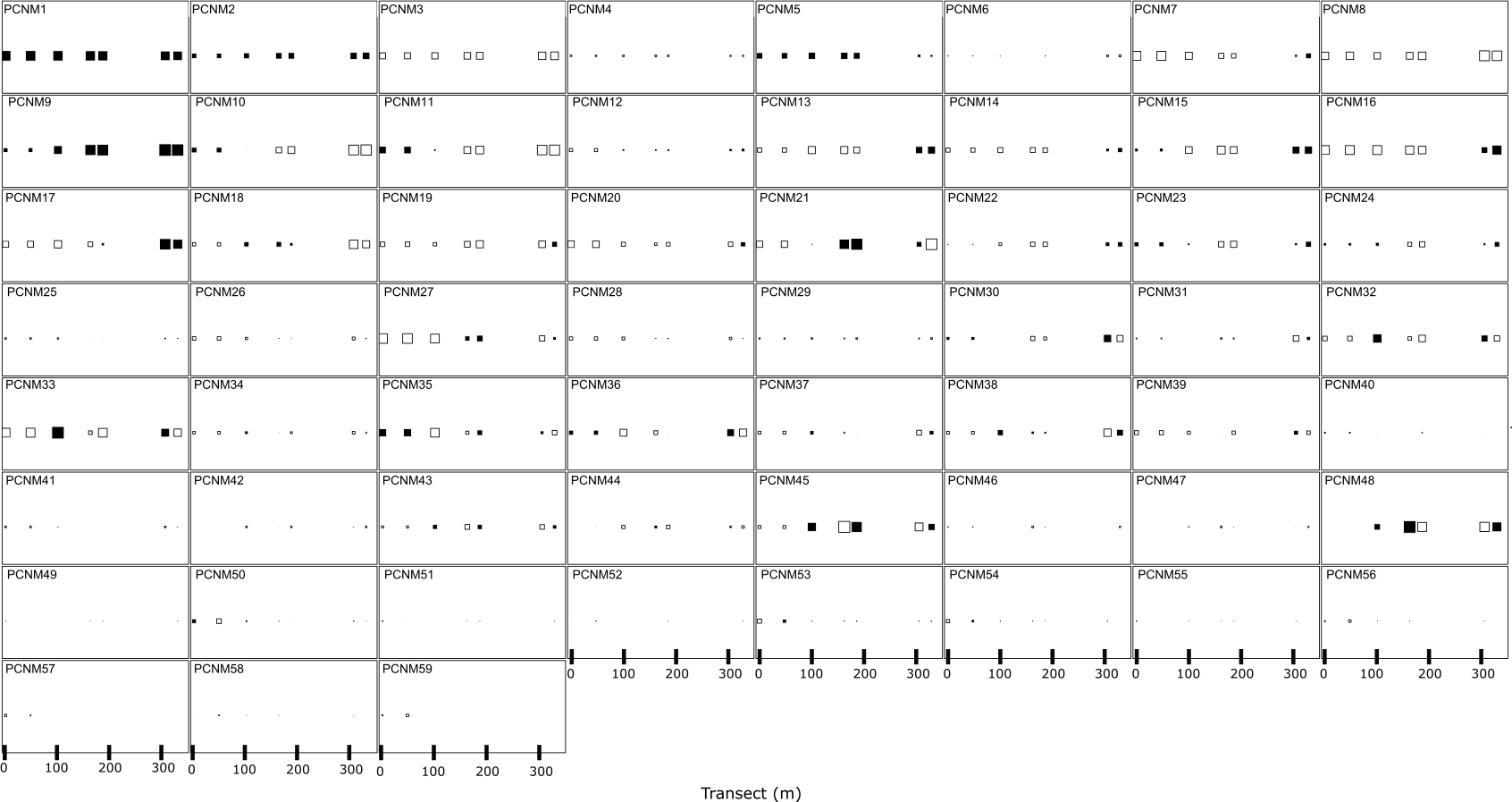
**
