# Supplementary material for: A Genome-Wide Association study in Arabidopsis thaliana to decipher the adaptive genetics of quantitative disease resistance in a native heterogeneous environment
Source: PLoS One. 2022 Oct 3;17(10):e0274561. doi: 10.1371/journal.pone.0274561 (PMC9529085; doi:10.1371/journal.pone.0274561)

**S4 Figure. A polygenic architecture underlying natural genetic variation of total seed production within each micro-habitat for which disease index was significantly heritable.** Manhattan plot of the Lindley process (local score method with a tuning parameter ξ = 2). The x-axis indicates the physical position of the 981,617 SNPs along the five chromosomes. The dashed lines indicate the minimum and maximum of the five chromosome-wide significance thresholds.


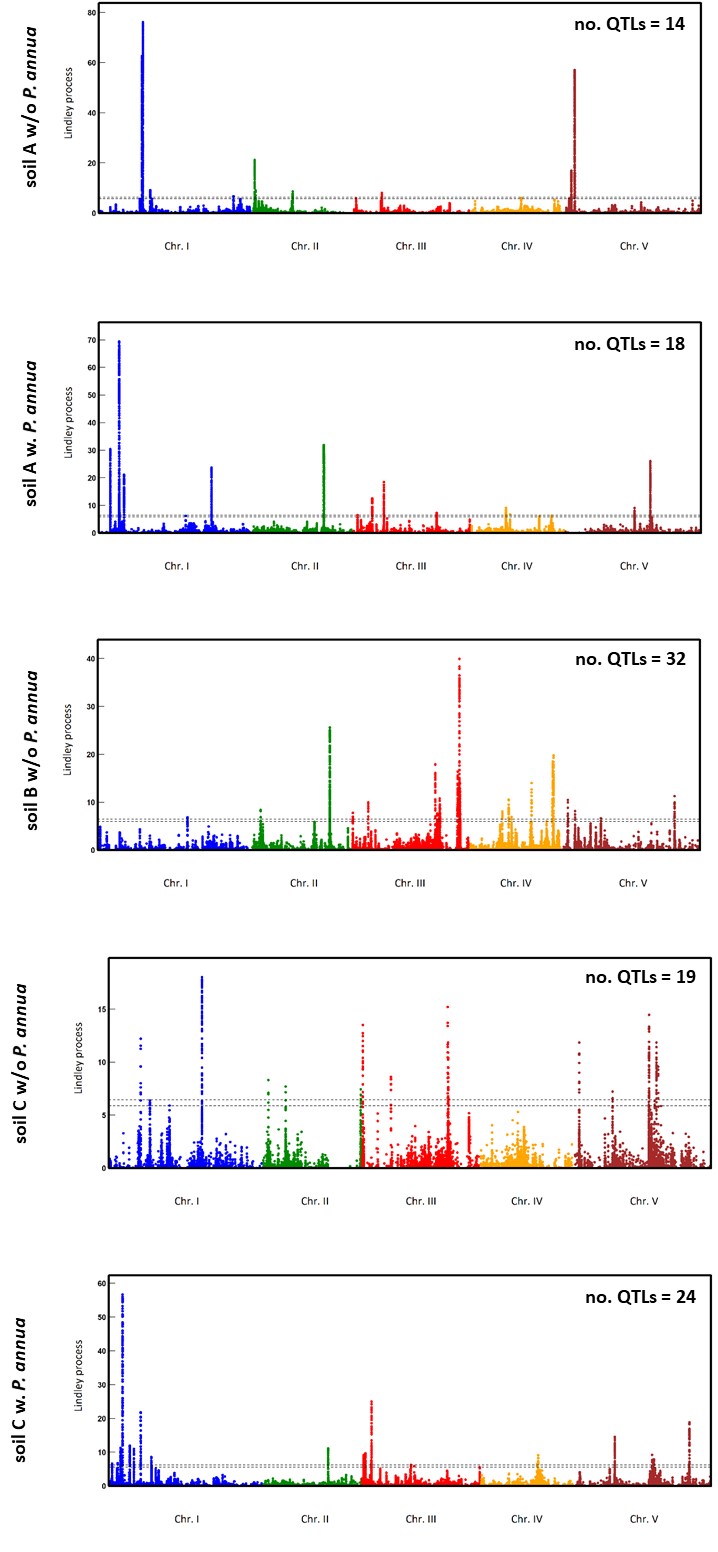

Supplement: S4 Fig — Manhattan plot of the Lindley process (local score method with a tuning parameter ξ = 2). The x-axis indicates the physical position of the 981,617 SNPs along the five chromosomes. The dashed lines indicate the minimum and maximum of the five chromosome-wide significance thresholds. (DOCX) [file pone.0274561.s008.docx]
