# Supplementary material for: A Genome-Wide Association study in Arabidopsis thaliana to decipher the adaptive genetics of quantitative disease resistance in a native heterogeneous environment
Source: PLoS One. 2022 Oct 3;17(10):e0274561. doi: 10.1371/journal.pone.0274561 (PMC9529085; doi:10.1371/journal.pone.0274561)

**S5 Figure. A variable architecture underlying natural genetic variation of total seed production within each micro-habitat for which disease index was significantly heritable.** UpSet plot illustrating the number of candidate genes that were either specific to a single micro-habitat (i.e. single black dots) or common between two micro-habitats (i.e. black dots connected by a solid line). ‘w/o’: absence of *P. annua*, ‘w.’: presence of *P. annua*. For each micro-habitat, the number of candidate genes identified by GWA mapping (colored bars) corresponds to the sum of the numbers of candidate genes above the grey bars for which dots are present. For instance, the total number of candidate genes identified by GWA mapping for the micro-habitat ‘soil C w:O *Poa annua*’ is 55, which corresponds to the sum of the values 51, 3 and 1.


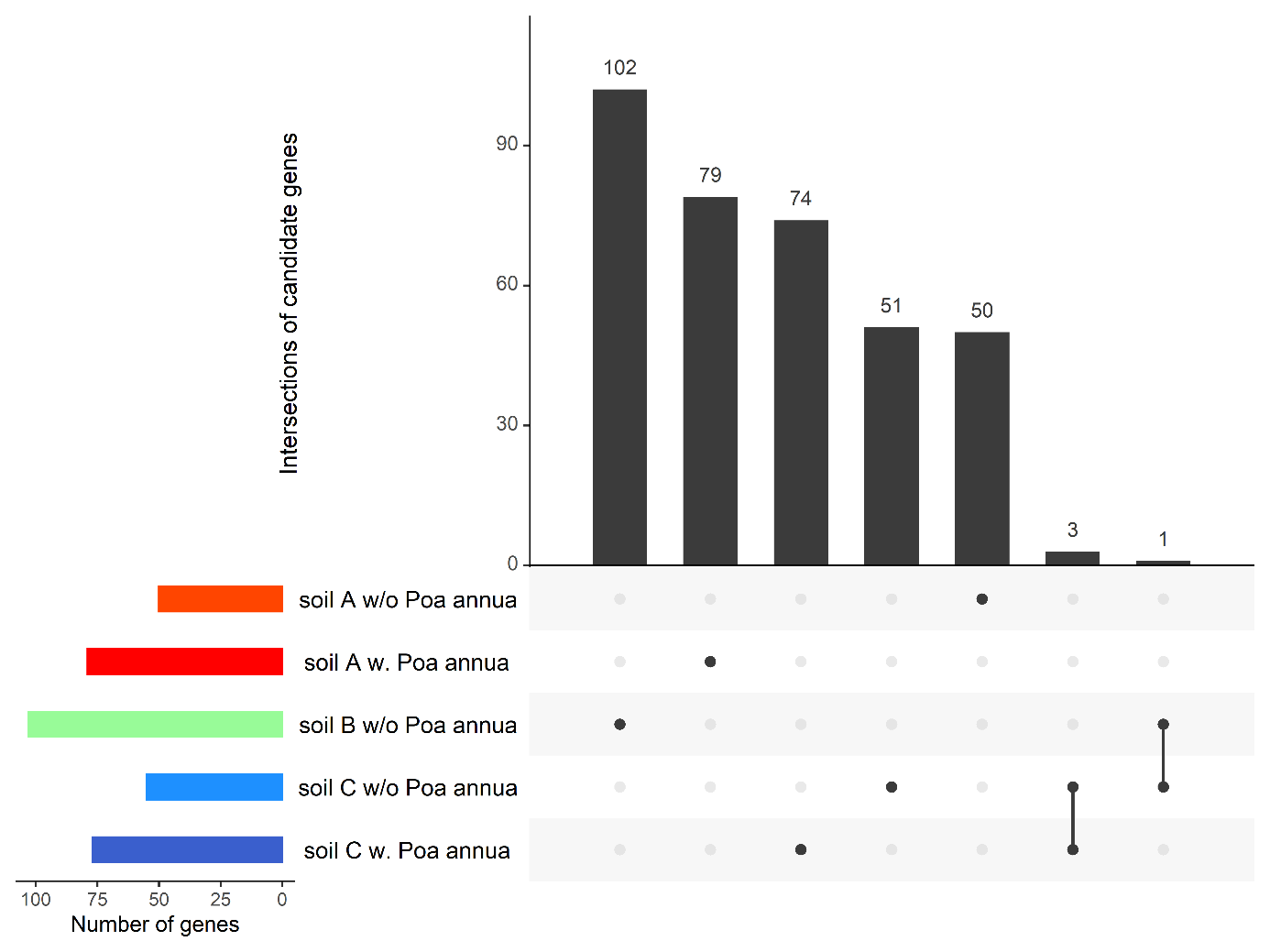

Supplement: S5 Fig — UpSet plot illustrating the number of candidate genes that were either specific to a single micro-habitat (i.e. single black dots) or common between two micro-habitats (i.e. black dots connected by a solid line). ‘w/o’: absence of P. annua, ‘w.’: presence of P. annua. For each micro-habitat, the number of candidate genes identified by GWA mapping (colored bars) corresponds to the sum of the numbers of candidate genes above the grey bars for which dots are present. For instance, the total number of candidate genes identified by GWA mapping for the micro-habitat ‘soil C w:O Poa annua’ is 55, which corresponds to the sum of the values 51, 3 and 1. (DOCX) [file pone.0274561.s009.docx]
