# Supplementary material for: A Genome-Wide Association study in Arabidopsis thaliana to decipher the adaptive genetics of quantitative disease resistance in a native heterogeneous environment
Source: PLoS One. 2022 Oct 3;17(10):e0274561. doi: 10.1371/journal.pone.0274561 (PMC9529085; doi:10.1371/journal.pone.0274561)
Supplement: S6 Fig — (A) soil A in absence of Poa annua. (B) soil A in presence Poa annua. (C) soil B in absence of Poa annua. (D) soil C in absence of Poa annua. (E) soil C in presence Poa annua. Manhattan plot of the Lindley process (local score method with a tuning parameter ξ = 2). The x-axis indicates the physical position of the 981,617 SNPs along the five chromosomes. The dashed lines indicate the minimum and maximum of the five chromosome-wide significance thresholds. (DOCX) [file pone.0274561.s010.docx]

**S6 Figure. Comparison of the polygenic architecture underlying natural genetic variation of disease index and total seed production within each micro-habitat.** (A) soil A in absence of *Poa annua*. (B) soil A in presence *Poa annua*. (C) soil B in absence of *Poa annua*. (D) soil C in absence of *Poa annua*. (E) soil C in presence *Poa annua*. Manhattan plot of the Lindley process (local score method with a tuning parameter ξ = 2). The x-axis indicates the physical position of the 981,617 SNPs along the five chromosomes. The dashed lines indicate the minimum and maximum of the five chromosome-wide significance thresholds.

**
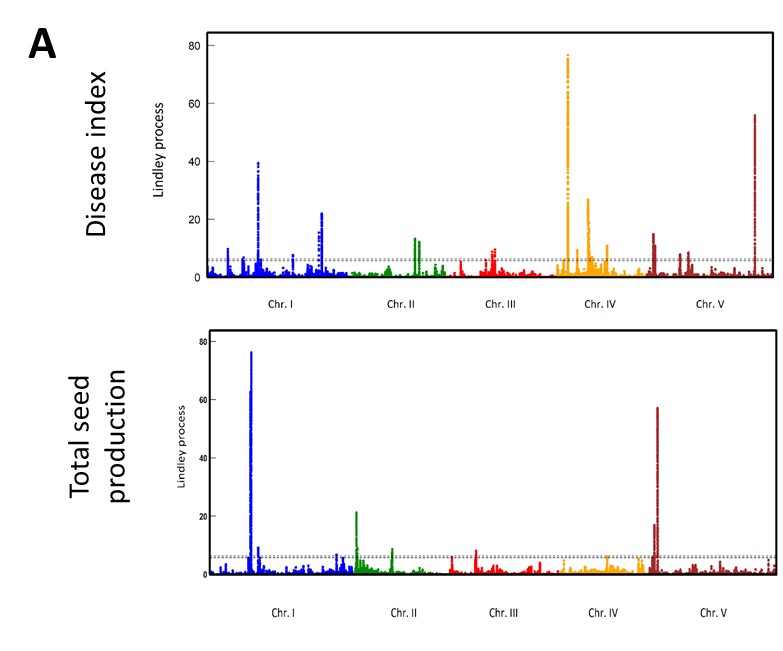
**

**S6 Figure (continued)**

**
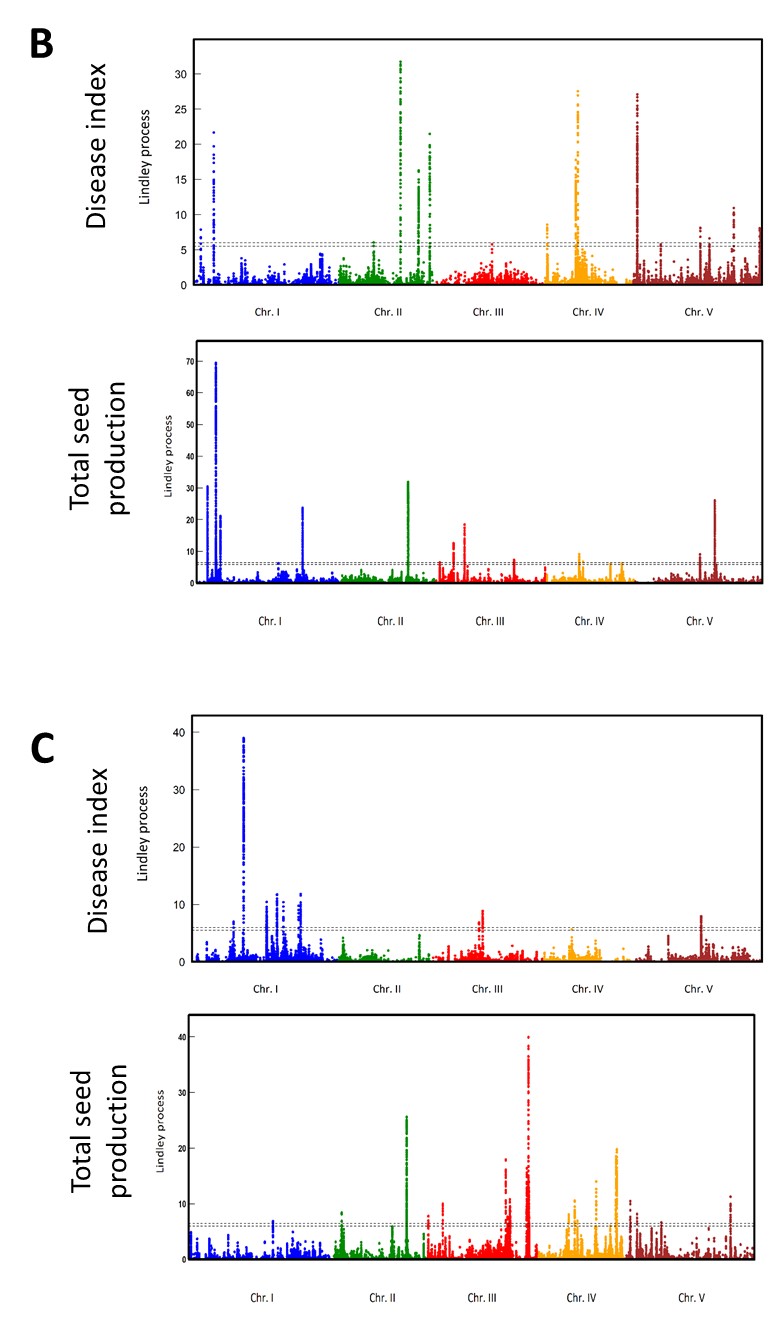
**

**S6 Figure (continued)**

**
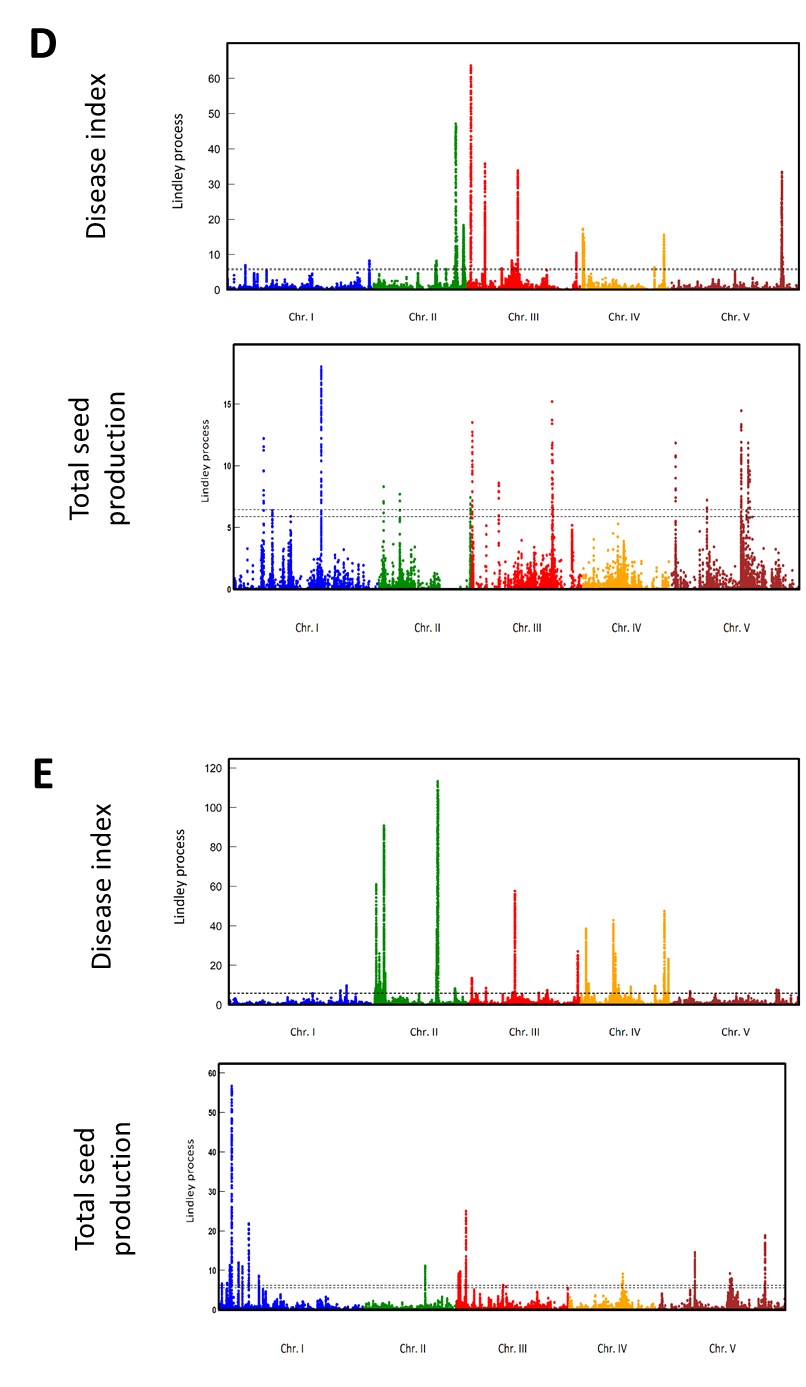
**
